# Shoulder arthroplasty in osteoarthritis: current concepts in biomechanics and surgical technique

**Published:** 2013-05-06

**Authors:** G Merolla, G Nastrucci, G Porcellini

**Affiliations:** * Unit of Shoulder and Elbow Surgery, D. Cervesi Hospital, Cattolica – Italy; ** Unit of Orthopedics, Campolongo Hospital, Campolongo - Italy; † Laboratory of Biomechanics “Marco Simoncelli”, D. Cervesi Hospital, Cattolica – Italy

**Keywords:** shoulder, osteoarthritis, prostheses, surgical technique

## Abstract

Shoulder arthroplasty is a technically demanding procedure to restore shoulder function in patients with severe osteoarthritis of the glenohumeral joint. The modern prosthetic system exploit the benefits of modularity and the availibility of additional sizes of the prosthetic components. In this paper we describe the biomechanics of shoulder arthroplasty and the technique for shoulder replacement including total shoulder arthroplasty (TSA) with all-polyethylene and metal-backed glenoid component, humeral head resurfacing and stemless humeral replacement.

## I. INTRODUCTION

**Fig. 1:**
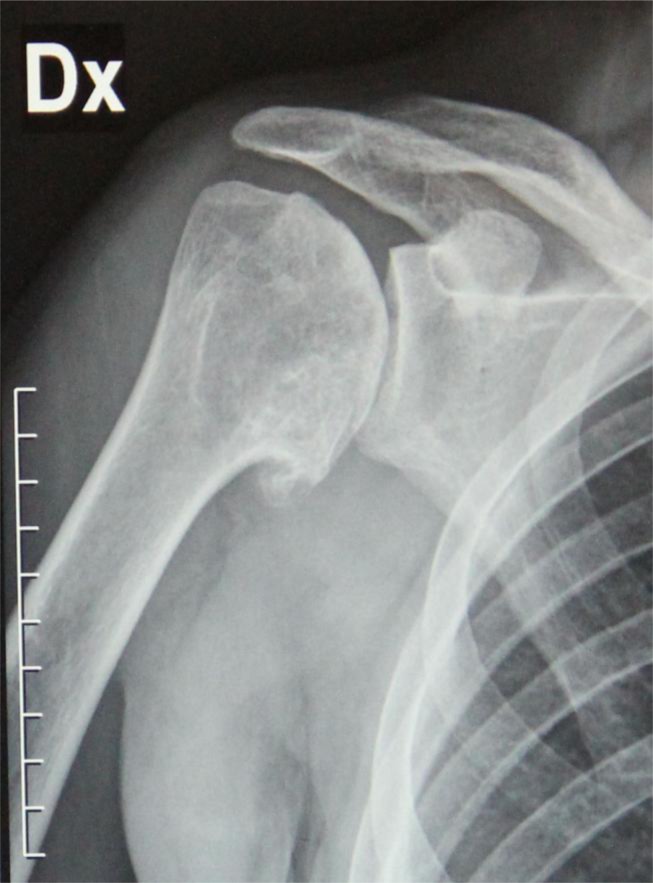
X-ray in true AP view shows severe gleno-humeral osteoarthritis with complete obliteration of joint space, glenoid erosion, and the humeral and glenoid osteophytes.

Shoulder arthroplasty remains the standard treatment to restore shoulder function and improve patient’s quality of life in severe arthritis of the glenohumeral joint ( Fig. 1 ). Charles Neer [ 1 ] firstly reported satisfactory results with humeral replacement, but a long term evaluation showed that cohort of the patients continued to complain of shoulder pain, slow strengh recovery and prolonged weakness after hemiarthroplasty. These complications were attributed to implant mobilization [ 2 ] , glenoid erosion [ 3 ] and rotator cuff deficiency [ 4 ] . Consequently, a polyethylene glenoid component was introduced to reduce the risk of prostheses failure and related worsening in quality of life [ 2 ] . The modern prosthetic system exploit the benefits of modularity and the availibility of additional sizes of the prosthetic components. In this paper we describe the biomechanics of shoulder arthroplasty and the technique for shoulder replacement including total shoulder arthroplasty (TSA) with all-polyethylene and metal-backed glenoid component, humeral head resurfacing and stemless humeral replacement. All the patients gave informed consent prior to being included in the study. As this study was a review with standard of care, local ethics committee authorization was not required. The study was performed in accordante with the ethical standards of the 1964 Declaration of Helsinky as revised in 2000.

## 2. PROTSTHESES BIOMECHANICS

**Fig. 2:**
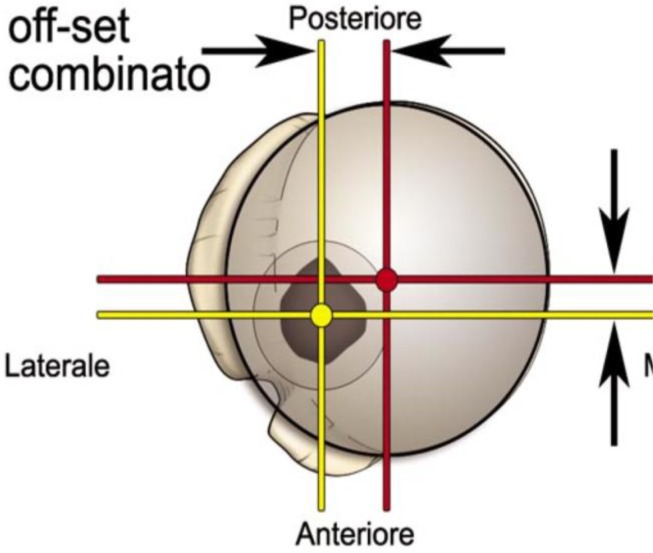
Combined humeral head offset.

**Fig. 3:**
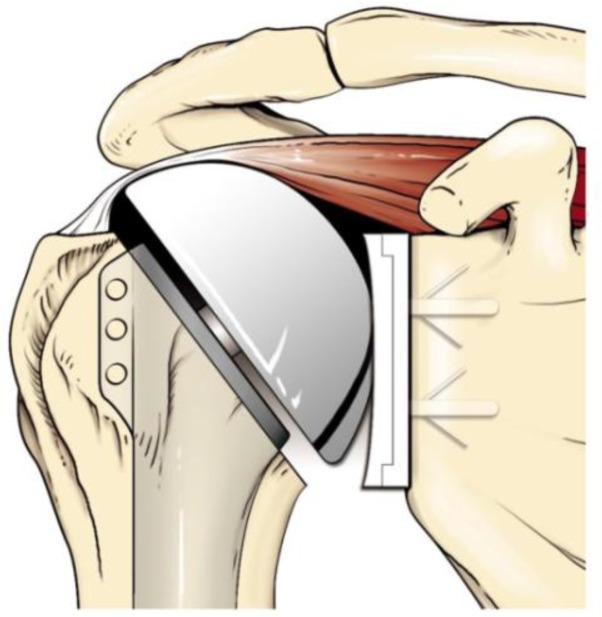
Overstaffing of the humeral head due to the excessive protrusion above the greater tuberosity.

The main goals of shoulder prostheses are pain alleviation and full functional recovery. Satisfactory results of replacement depends on: 1) prosthetic reproduction of a normal bone morphology ( *shape of the humeral epiphysis and the glenoid thatare identical to the normal structures in size, orientation, centres of rotation, lever arm of the cuff tendons and of the deltoid muscle* ); 2) optimum restoration of capsular tension to remove the asymmetric constraints induced by changes in capsule volume; 3) restoration of the stabilizing and motor function of the muscle. The main geometric parameters of a shoulder arthroplasty include as follow: neck inclination, humeral head diameter and thickness, humeral head height, humeral head retroversion, medial and posterior head offsets, acromion-humeral distance. The cervicodiaphyseal angle [ 5 ] is most often 135° ± 5°. Prostheses are usually designed with a fixed angle of 130°–135° and the instrumentations perform head osteotomy at that angle. The diameter of the humeral head [ 6 ] varies widely from 38 to 58 mm (median 46 mm). Degenerative diseases altering the spherical shape so the prosthetic head diameter often cannot be determined. The component’s diameter is thus chosen at the time of trial reduction based on other parameters with special regards to the height of the hemisphere that it has been seen to have broad linear relationship with the diameter of the head. In all humeri the superior edge of the head protrudes above the superior edge of the greater tuberosity by 2–5 mm [ 7 ] . When the head component is positioned under the edge of the greater tuberosity, the joint’s instantaneous centre of rotations descends, resulting on reduced lowering of the humeral head and increased tension in adduction, and signally, in early, painful subacromial impingement. On the other hand, a head protruding excessively above the greater tuberosity induces increased tension on the cuff (“overstuffing”) ( Fig. 2 ). The humeral head is retroverted with respect to the coronal plane. The angle of retroversion is the subtended between the epicondylar axis and the central axis of the humeral head. Its median values is 20° and it is proportional to the angle of retroversion of the scapula which instead is widely variable (0°–60°). Small errors in head retroversion do not significally influence the tension of the caspuloligamentous system nor the instantaneous centre of rotation; an excessive retroversion may induce posterior head subluxation in case of a posterior cuff tear, whereas an insufficient retroversion may cause subscapularis impingement. The centre of the head does not lies on the diaphyseal humeral axis, but is displaced both in the coronal and the transverse planes. In the coronal axis the offset ranges from 2 mm to 12 mm (median 7 mm) ( *medial and lateral offset* ) ( Fig. 3 ); lower values results in a looser capsuloligamentous complex, while excessive values produce overstuffing and possible joint stiffness. The centre of the head lies 0–10 mm (median 4 mm) posterior to the diaphyseal axis ( *posterior humeral head offset* ) ( Fig. 3 ) [ 8 ] ; if this features, and the instantaneous centre of rotation, move anteriorly induce an abnormal contact with the glenoid and abnormal pressure on the subscapularis. The space between humeral head and acromion is ca 2 cm. A wider space reduce muscle tension and produce loss of strenght in elevation while a narrower spacer result in a stiffer joint and possibly subacromial impingement.

### 

#### Prostheses design and components

**Fig. 4:**
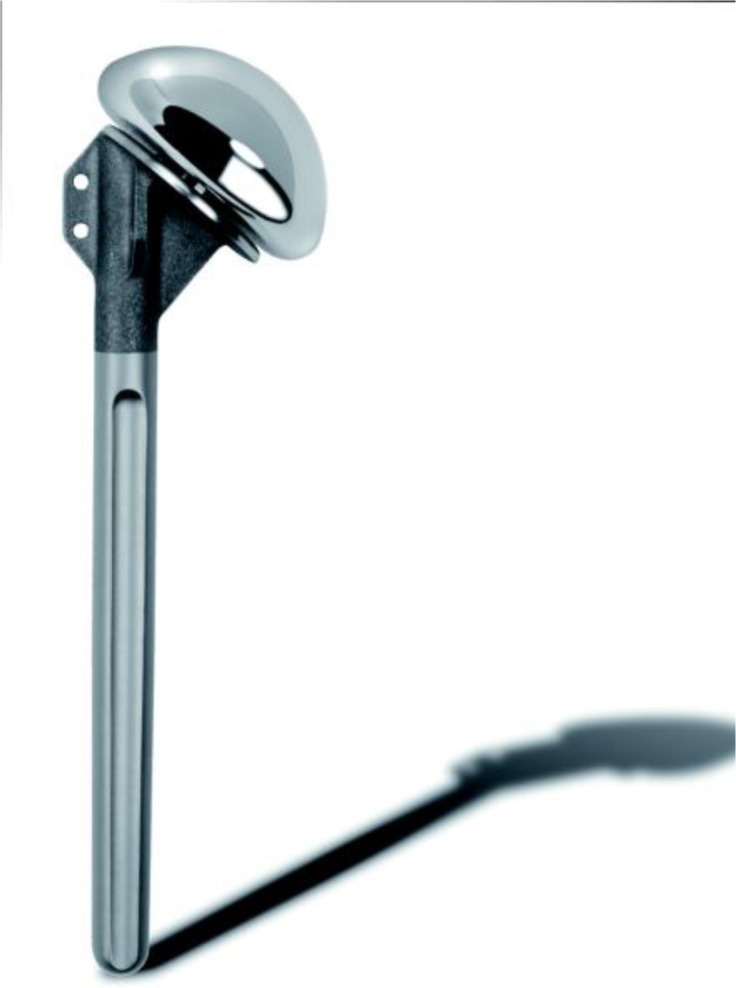
Monoblock humeral stem (Zimmer, Warsaw, IN - USA).

**Fig. 5A–D:**
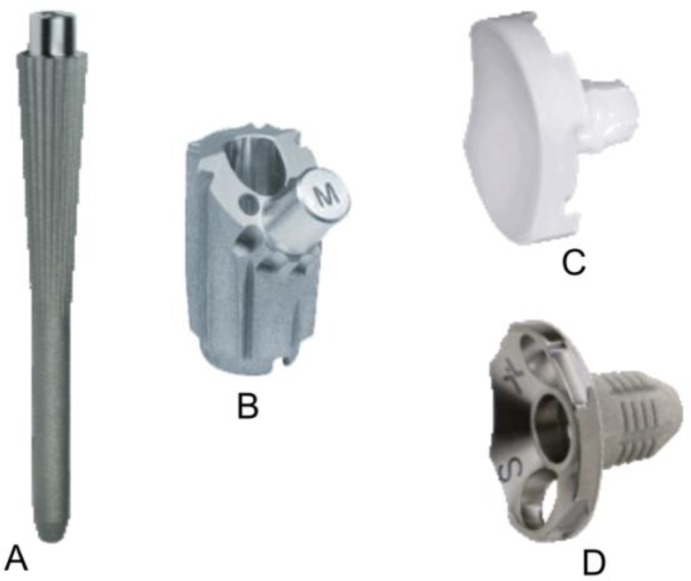
Humeral stem (A), humeral body (B), metal-backed glenoid component (C) and polyethylene liner of a modular humeral component (LIMA, San Daniele del Friuli - Italy).

**Fig. 6:**
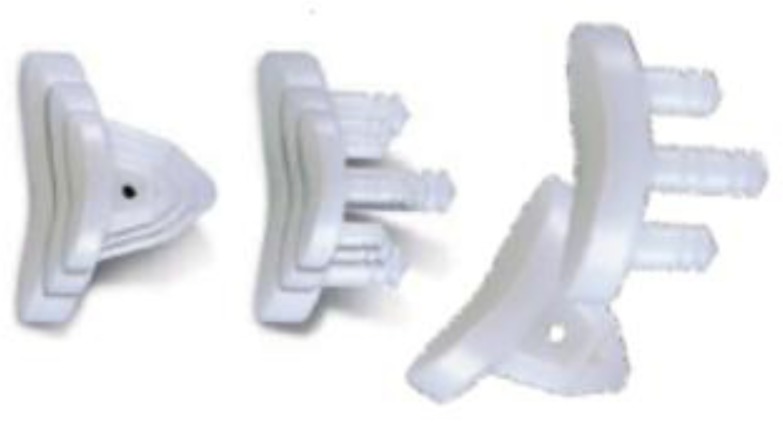
Keeled and pegged polyethylene glenoid component (Zimmer, Warsaw, IN - USA).

**Fig. 8:**
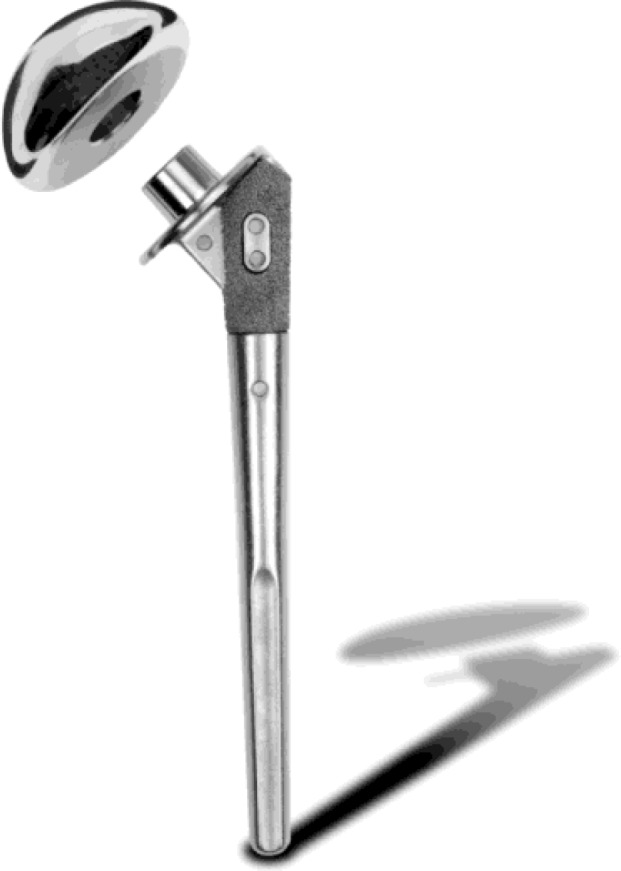
TMT humeral component (Zimmer, Warsaw, IN - USA).

Anatomical total shoulder arthroplasty make use of a unconstrained prostheses including monoblock ( Fig. 2 ) or modular ( Fig. 3A –B) humeral components and cemented all-polyethylene ( Fig. 4 ) or metal-backed glenoid component ( Fig. 5A –B). The last generation of glenoid component includes implants using trabecular metal technology (TMT ^®^ ) ( Fig. 6 ) [ 9 ] . Polyethylene glenoid prostheses are available with keeled and pegged models ( Fig. 4 ). The technique of shoulder arthroplasty requires a durable fixing of the humeral component in the proximal part of the humerus. This fixation is accomplished by the insertion of the component stem into a medullary canal that has been reamed to the stem diameter and the use of cement for fixation or a press-fit component for tissue ingrowth [ 10 , 11 ] . As for the glenoid a TMT humeral component enabling the healing of the humeral fractures is available ( Fig. 8 ) [ 12 ] .

## 3. SURGICAL TECHNIQUE

**Fig. 9:**
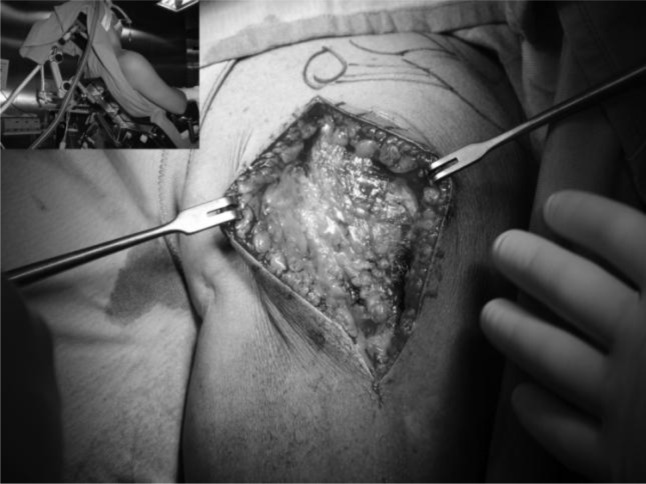
Skin incision and beach-chair position.

**Fig. 10:**
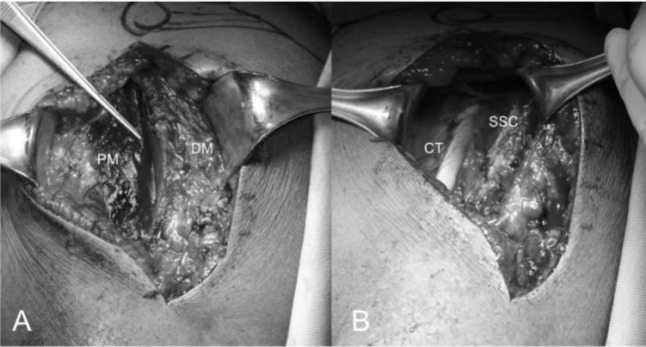
A–B: Deltopectoral interval with the cephalic vein along the edge of the pectoralis major (A), conjoint tendon and subscapularis tendon. PM: pectorali major; DM: deltoid muscle; CT: conjoint tendon; SSC: subscapularis

**Fig. 11:**
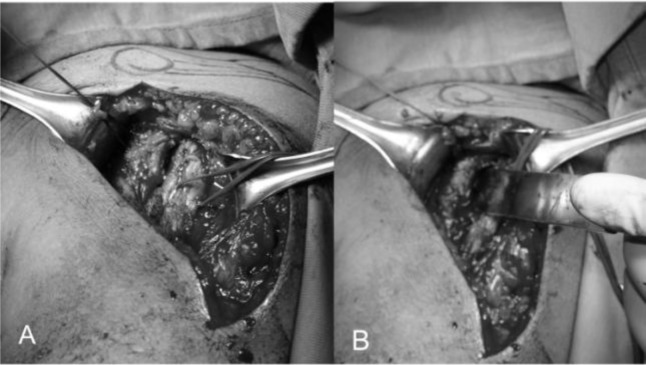
A_B: Long head of the biceps tendon in the bicipital grove (loop laterally) and subscapularis (suture marker medially) (A), lesser tuberosità osteotomy (B).

**Fig. 12:**
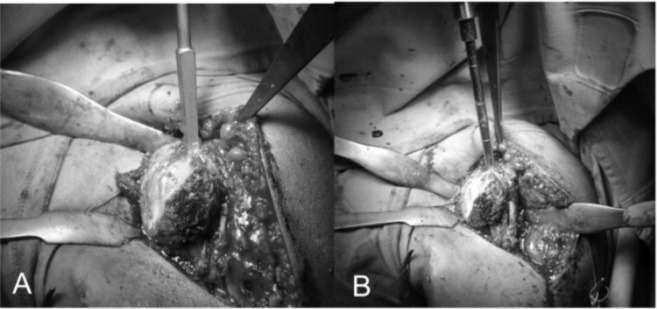
Complete exposure of the humeral head that is perforated through the “hinge point” (A). Graduated driving enter the medullary canal to prepare the head cutting (B).

The operation is performed with the patient under general anesthesia associated with interscalene block to have a better control of intraoperative bleeding and perioperative pain. The patient was placed in the beach chair position ( Fig. 9 ), with the upper part of the body raised 30 to 40 degrees with the head on a headpiece and the scapula hold forward. We used a standard delto-pectoral approach. We marked the skin landmarks and the line of the incision, we place the arm in 30° of abduction and we begin the cut from the clavicle down across the tip of the coracoid and continued in a straight line to the anterior border of the the deltoid insertion ( Fig. 9 ). We dissect the subcutaneous tissue from the deltoid fascia and we expose the deltoid and pectoralis major muscles. We identify the interval between the deltoid and pectoralis major muscle with the cephalic vein that is retracted laterally with the deltoid ( Fig. 10 A ). The clavipectoral fascia is incised along the lateral border of the coracobrachialis tendon ( Fig. 10B ). At this stage a better exposure will be obtained by cutting the proximal 2 cm of the pectoralis major insertion. We check for the long head of biceps in the bicipital groove that is tenotomized ( Fig. 11 A ). We identifie the subscapularis tendon that sometimes can be degenerated and retracted and with the arm in external rotation we check its superior and inferior borders and the anterior humeral circumflex vessels (“the three sisters”) that goes around inferiory. The tendon is isolated with non-absorbable sutures and the lesser tuberosity with subscapularis tendon is osteotomized ( Fig. 11 B). The dissection proceed superiorly, from the base of the coracoid to the subacromial space, anteriorly and inferiorly carefully removing the degenerate capsule. We explore the subacromial space, saving the coracoacromial ligament, we pass a suture on the medial margin of the supraspinatus tendon to have a tendon mark in case we decide to close the rotator interval and we medially retract the subscapularis muscle to expose th joint. We put the Hohmann levers and we begin the maneuvers to dislocate the humeral head that are facilitated by a movement of the arm in adduction, extension and external rotation. At this stage it is necessary to completely remove the inferior “goat beard” osteophyte to have the complete exposure of the humeral head ( Fig. 12 A ).

### 

#### Humeral exposure: tips and tricks [ 13 ]

We prefer to take the cephalic vein laterally because the most tributaries derives from the deltoid muscle. It is common to find some small tributaries veins cross the upper part of the delto-pectoral interval that need to be cauterized to avoid troublesome bleeding. Dissection under the deltoid muscle must be developed using the electrocautery close to bone to avoid njuries to the axillary nerve. The tip of the coracoid identify the origin of the conjoined tendon as a landmark to begin the incision of the clavipectoral fascia laterally and proximally to the anterior margin of the coracoacromial ligament that should be preserved to prevent the risk of anterosuperior subluation of the head prostheses. At this stage is recommended to palpate the axillary and musculocutaneous nerves to minimize the risk of injuries during the dissection or retraction. When the subscapularis is detached with the lesser tuberosity (“flake osteotomy”) the arm should be placed slightly abducted and internally rotated of 40° for an adequate osteotomy. Posterior capsular should be released using strong scissors to allow the arm to be externally rotated and prepared for humeral head resection. During humeral exposure we suggest to use a large retractor in the glenohumeral joint, a blunt Hohmann under the deltoid in the subacromial space and a small Hohmann at the inferior humeral neck with the retractor in contact with the bone to keep a safe distance from the axillary nerve.

#### Glenoid exposure: tips and tricks [ 13 ]

The exposure of the glenoid is the most difficult step in shoulder arthroplasty. The relaxation of the posterior and superior capsule allow more posterior humeral displacement that can be obtained having the arm with the osteotomy surface as parallel as possible to the glenoid surface; then the arm is adjusted to have the maximum exposure. The fukuda retractors and two small Hohmann retractors, one superiorly and one anteroinferiorly provide an excelent glenoid exposure. The capsule is released anteriorly and inferiorly past the 6 o’clock position; some authors suggest to left the subscapularis attached for tendon reinforcement [ 13 ] . If posterior subluxation is preoperatively found, some authors recommend to preserve posterior capsule [ 13 ] to avoid posterior instability, but this step is not common in our unit. During glenoid replacement, the central hole must be perpendicular to the glenoid surface and it may be helpful to use a reamer without a tip to preferentially ream anteriorly to correct the version [ 13 ] .

### Humeral replacement

**Fig. 13:**
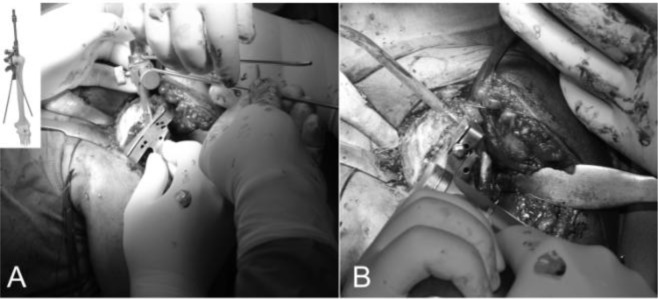
A–B: Mask for the humeral head and position of the guides to adjust humeral head osteotomy at 30° of retroversion (A). The cutting performed along the anatomical neck (B).

**Fig. 14:**
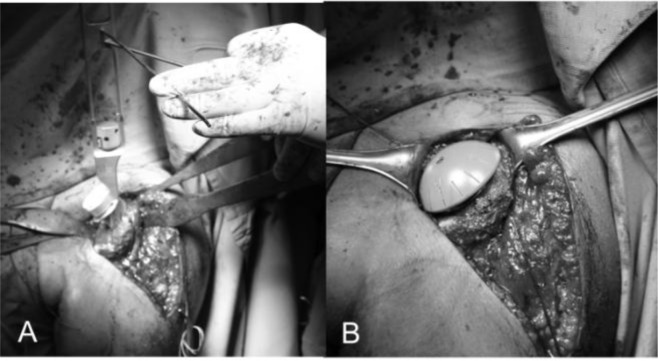
A–B: Trial stem inside and checking for the correct version (A) and humeral head trial (B)

For the preparation of the humerus must be removed all osteophytes present along the anatomical neck. With a tip perforates the humeral head at its highest point 1 cm superior-medial to bicipital groove, the so-called “hinge point” ( Fig. 12 A ) and enter the medullary canal through a graduated driving, which then can be mounted on the mask for cutting ( Fig. 12 B). Osteotomy of the head is carried out exactly at the anatomical neck, respecting the correvct degree of retroversion (30°) ( Fig. 13 A–B ). We bore the channel with a hand drill gradually increasing the diameter to create a recess adapted to accommodate the implant. We insert the trial stem carefully observing the degree of retroversion: with the arm in neutral rotation the Morse taper of the stem should be oriented toward the center of the glenoid ( Fig. 14 A ). After positioned the stem we choose the prosthetic head closest to the original humeral anatomy. We put the head on the chosen trial stem and we correct the off-set by rotating the eccentric head giving uniform coverage to the humeral neck without creating abnormal stresses on the rotator cuff ( Fig. 14 B). We perform the reduction maneuver cautiously, we assesses the stability and the ROM of the implant that should be not lesser than 90 ° in internal rotation, 120 ° in elevation and 30 ° in external rotation. Then we redislocate the shoulder, we remove the trial head leaving the stem inside to reduce the bleeding and we pass to the glenoid phase.

### Glenoid replacement

#### Cemented all-polyethylene component

**Fig. 15A–B:**
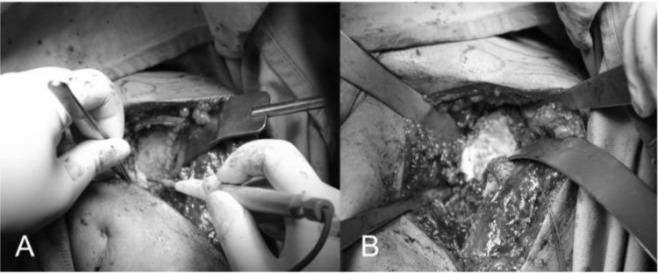
Glenoid exposure with Fukuda retractor (A) and curved retractor (B). The capsule is excised circumferentially.

**Fig. 17:**
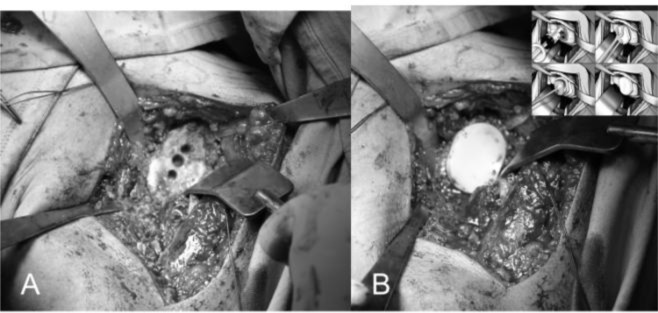
A–B: Preparation of the glenoid with the three holes (A) for the glenoid trial. Cemented pegged glenoid component implanted using a standard technique (B) (reprint with permission by Porcellini et al. “Shoulder replacement in osteoarthritis” p. Bologna, Italy: Timeo editore 2005) (see the text).

The replacement of the glenoid is technically more complicated and difficult than the humerus. We begin putting the limb at 70–90° of abduction, in external rotation and in moderate flexion, then we place a Fukuda retractor on the glenoid to posteriorly and inferiorly subluxate the humeral head for the better exposure of the glenoid ( Fig. 15 A ). The exposure of the posterio-inferior glenoid border can be facilitated by the placement of a curved retractor ( Fig. 15 B ). We remove the capsule from the edges of the glenoid and the entire labrum at 360°, we define the orientation of the articular surface of the glenoid that is regulated and measured and we create a first center hole to drill the surface with a reamer and expose the subchondral bone in order to obtain an omogeneous surface for an effective bone-prostheses bond ( Fig. 6 A–B0 ). The reaming is a very delicate moment for two reason: 1) you can correct the orientation of the glenoid defects, 2) you must take care not to remove an excessive amount of subchondral bone to avoid weakning of the glenoid bone with risk of fractures. At this point we create with the guides and the appropriate forms the holes to accomodate the prostheses. We proceed with the creation of the other two holes for the trial component and we test the intrinsic instability ( Fig. 17 A ). Verified the final size of the glenoid component we begin the cementing procedure that follow a standard technique ( Fig. 17 B ). We remove the glenoid trial, we make a generous washing and then we inject the cement in the cuts for pegs using a 60 ml pressurized syringe, we impact the cement with a dedicated instruments, repeating the application in the holes with the syring and manually on the nack surface of the component, then the final glenoid prostheses (Zimmer, Warsaw, IN -USA) is impacted and kept under pressure waiting for the consolidation after which we accuarately remove the excess of cement ( Fig. 17 B ).

#### Metal-backed component

**Fig. 18:**
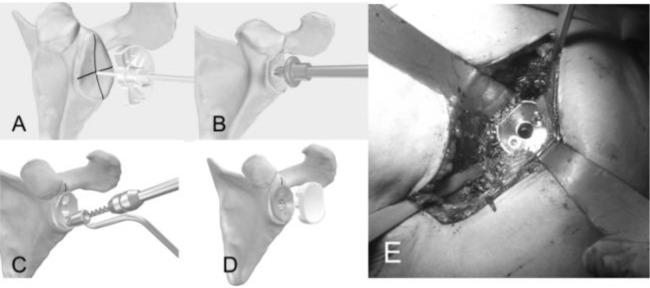
A–E: Preparation of the glenoid for metal-backed implant. Removing of the glenoid cartilage to expose the subchondral bone (A), glenoid drilling (B), metal-backed cementless glenoid component impacted in the central hole and screw fixation at 30° (C, E), insertion of the polyethylene liner (D) (reprint with permission by LIMA Corporate, San Daniele del Friuli – Italy).

**Fig. 19:**
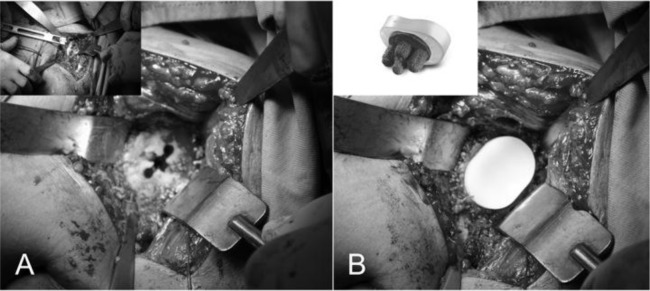
A–B: Preparation of the glenoid to insert a TMT component (A). TMT glenoid prostheses implanted without cement (B) (Zimmer, Warsaw, IN – USA).

We identify the centre of the glenoid tracing two orthogonal lines along the longitudinal and transversal axes with an electric cautery, the we insert a K wire (15 cm long, 2.5 mm diameter) into the bone for at least 25 mm orthogonal to the glenoid surface slightly off the centre ( Fig. 18 A ). We apply the glenoid reamer and remove the glenoid cartilage to expose the subchondral bone ( Fig. 18 A ). Follow on using the Small-R (Small-R metal back M-B) glenoid drill and insert until it comes to the end ( Fig. 18 B ); in case of larger peg use the glenoid drill to widen the hole. After choosing the size of the M-B cementless component we push it in the central hole with a positioner handle ensuring that the major axis of the implant conicides with largest axis of the glenoid ( Fig. 18 C ). We insert two screws and we fit them directing within 30° ( Fig. 18 C ); the two screws must be tightned simultaneously at the end to guarantee an otimal fixation of the metal in the bone ( Fig. 18 D–E ). Finally insert the polyethylene liner pushing with the thumb ( Fig. 18 D ). Alternatively a modern TMT ^®^ metal-backed glenoid (Zimmer, Warsaw, IN -USA) without screw fixation can be used to optimize the bone ingrowth and reduce the risk of glenoid failure ( Fig. 19 A–B ).

#### Final assembly of the prosthetic components

**Fig. 20:**
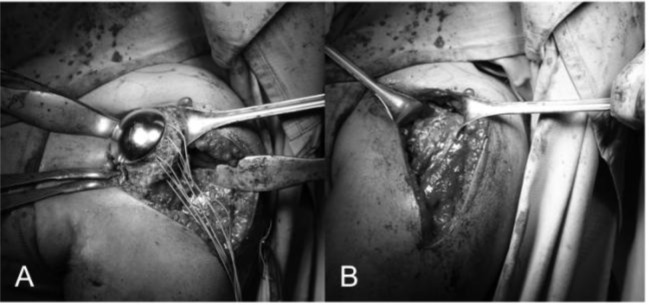
A–B: Final head prostheses uniformly covering the humeral neck and bone sutures for subscapularis reattachment (A). Subscapularis is reattached using bone to bone suture and the rotator interval is closed at its base (B).

Before implantation of the final humeral component we put again the trial head and we reduce the shoulder. We check the tension of the soft parts, the size, the offset of the head, the new articular relationship between the glenoid prostheses implanted and the ROM; we return the subscapularis to its bone insertion on the lesser tuberosity to assess the degree of tension. Assessed these parameters, we remove the humeral trial and we pass 4 or 5 bone sutures (flexidene # 4) in the neck of the humerus to fix the subscapularis ( Fig. 20 A ). In case we choose a cemented humeral prostheses, we insert the plug in the canal, we draw and we perform an accurate lavage. The cement is injected under pressure and we introduce the final stem with the correct version previously measured. We wait for the consolidation of the cement, we insert the trial head again to check once more the offset, the tension of the subscapularis, of the rotator cuff and the ROM. We remove the trial and we implant the final head prostheses (Zimmer, Warsaw, IN -USA) being sure to reproduce the offset previously assessed. We reduce the shoulder, we close the rotator interval to its base with reabsorble suture (ethibond #2) and we fix the subscapularis using a modified Mason-Allen stiches ( Fig. 20 B ). We repeat anterior and posterior drawer maneuvers to assess the stability of the prostheses and we evaluate the mobility achieved; we wash the area, we check the status of the axillary nerve and we place a subdeltoid drainage. We close the deep and surface layers, we place the arm in a sling and then we send the patient for the postoperative x-Ray control.

#### Resurfacing arthroplasty

**Fig. 21:**
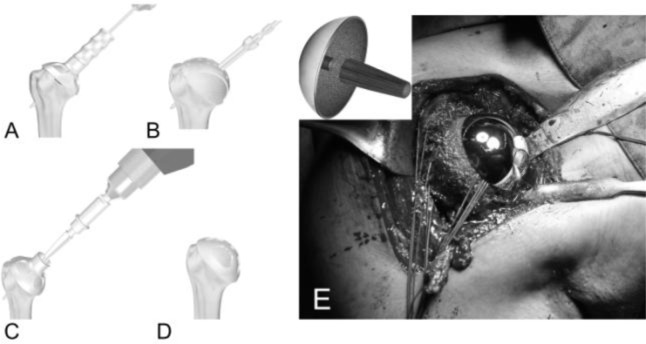
A–E: Humeral head reaming using a k wire as guide (A–B), drilling for the central hole (C), humeral head trial (D) and resurfacing prostheses with suture for bone to bone subscapularis reattachment (E) (reprint with permission by LIMA Corporate, San Daniele del Friuli – Italy).

Humeral head replacement is exposed as reported in the previous paragraphs. We locate the centre of the head using a k wire as guide ( Fig. 21 A ) and we ream with fully cannulated instruments system to restore humeral head shape and contour to allow a close fit of the final implant ( Fig. 21 B ). We drill the central hole for the tapering docking peg ( Fig. 21 C ), we place the trial head to choose the size ( Fig. 21 C ) and we fix the resurfacing head (LIMA, San Daniele del Friuli - Italy) having a Ti, plasma spray HA coating on their under side to aid fast osteointegration and resulting instability ( Fig. 21 D–E ). Glenoid can be replaced using a polyethylene component to obtain a total resurfacing arthroplasty.

#### Stemless humeral replacement

**Fig. 22:**
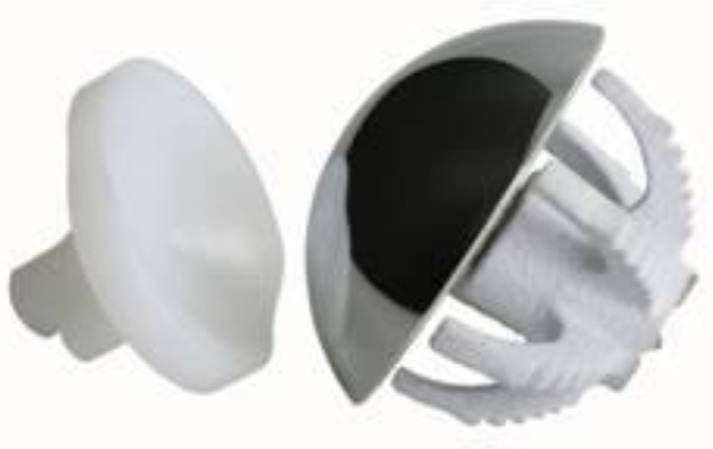
Stemless shoulder prostheses: note the “corolla” for the metaphyseal ingrowth in the cancellous bone and the polyethylene glneoid component for total shoulder replacement (TESS Biomet, Warsaw, IN – USA).

**Fig. 23:**
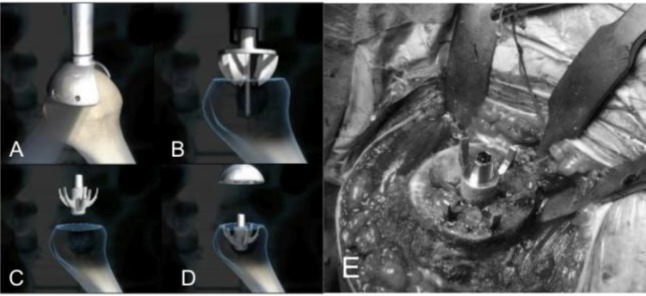
A–E: Surgical steps showing the cut of the humeral head at level of the anatomical neck (A), pin drilling trough the humeral template and puncher impactation (B) to insert the corolla (C) with the head resurfacing (D). Intraoperative image with the corolla pressed in the humeral neck (E).

**Fig. 24:**
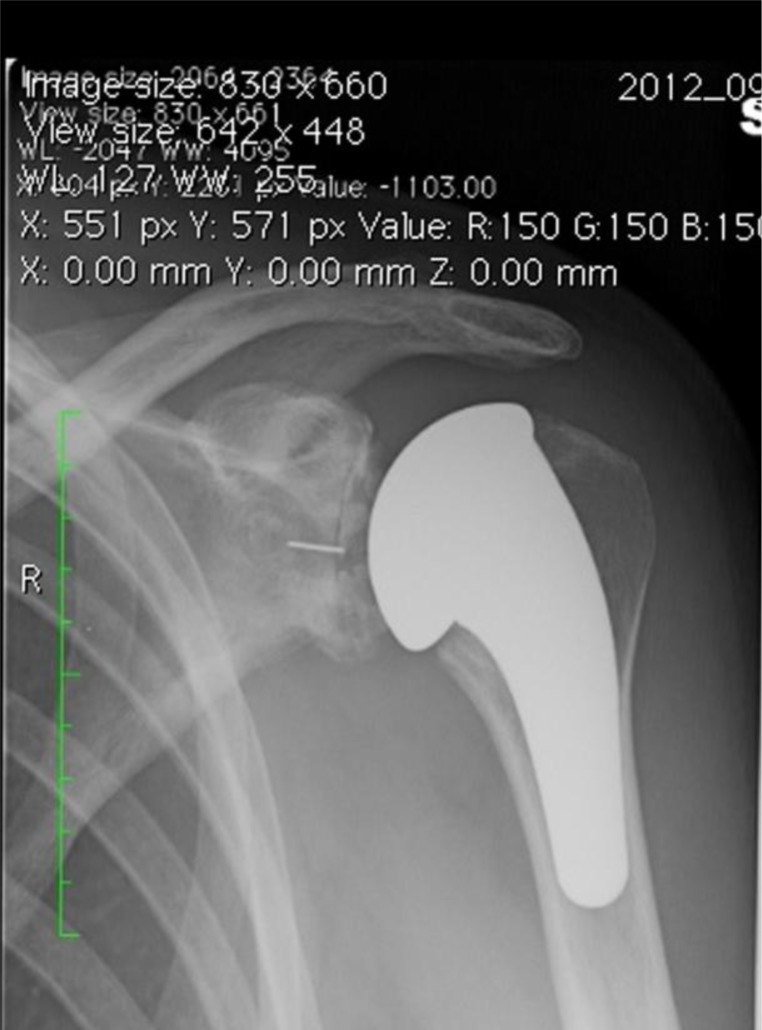
Postoperative X-ray of uncemented short stem TSA (Tornier, Inc, Montbonnot Saint Martin, France).

The stemless humeral prostheses (TESS ^®^ BIOMET, Warsaw, IN -USA) ( Fig. 22 ) represent the most modern system in the third generation of shoulder implants, developed to avoid the stem-related complications of shoulder implants [ 14 , 15 ] . A stable fixation is achieved using an ingrowth methaphyseal “corolla” pressed in the cancellous bone of the humeral neck ( Fig. 23 A–E ). After a complete exposure of the proximal humerus, we remove all the osteophytes to determine the size of the head, we cut the head at the level of the anatomical neck, a template is placed on the humerus to choose the size of the corolla, a pin is drilled through the centre of the humeral template and then the template is removed. A puncher is impacted over the guide pin that is removed and a trial head is placed on the punch, performing dynamic manouvers to evaluate height, stability and size of the final implant. In case of glenoid arthritis, a cemented polyethylene component can be implanted in a standard fashion ( Fig. 22 ). Short humeral stem have been recently introduced as alternative to the standard stem and stemless humeral component ( Fig. 24 )

#### Postoperative X-ray

**Fig. 25:**
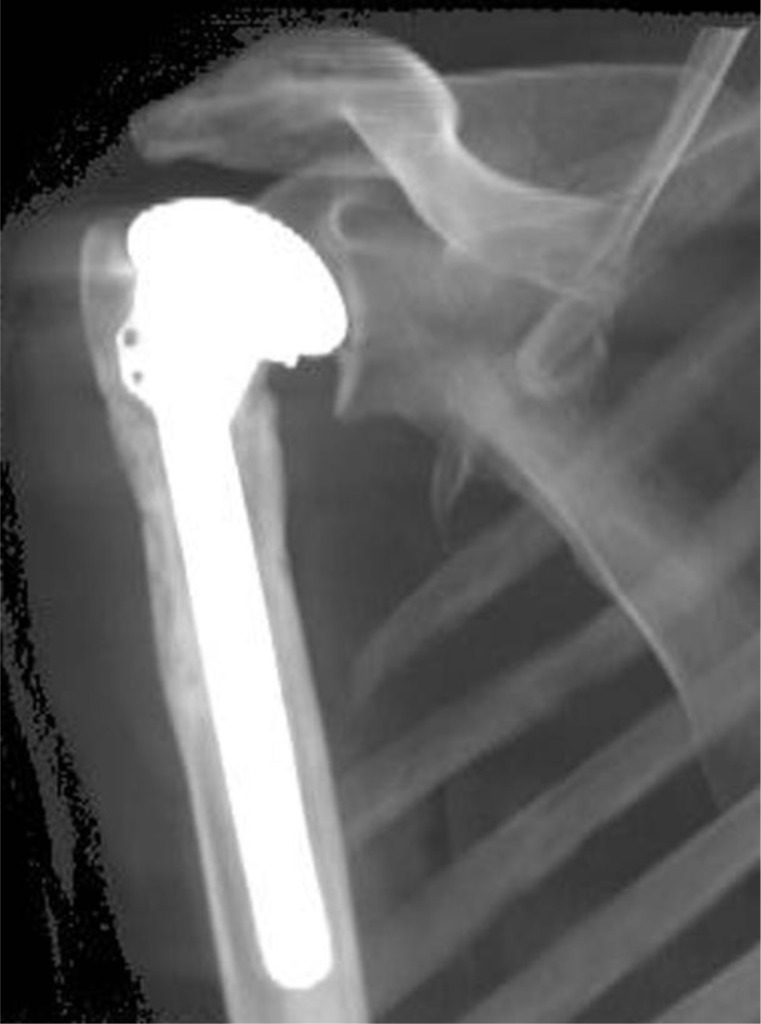

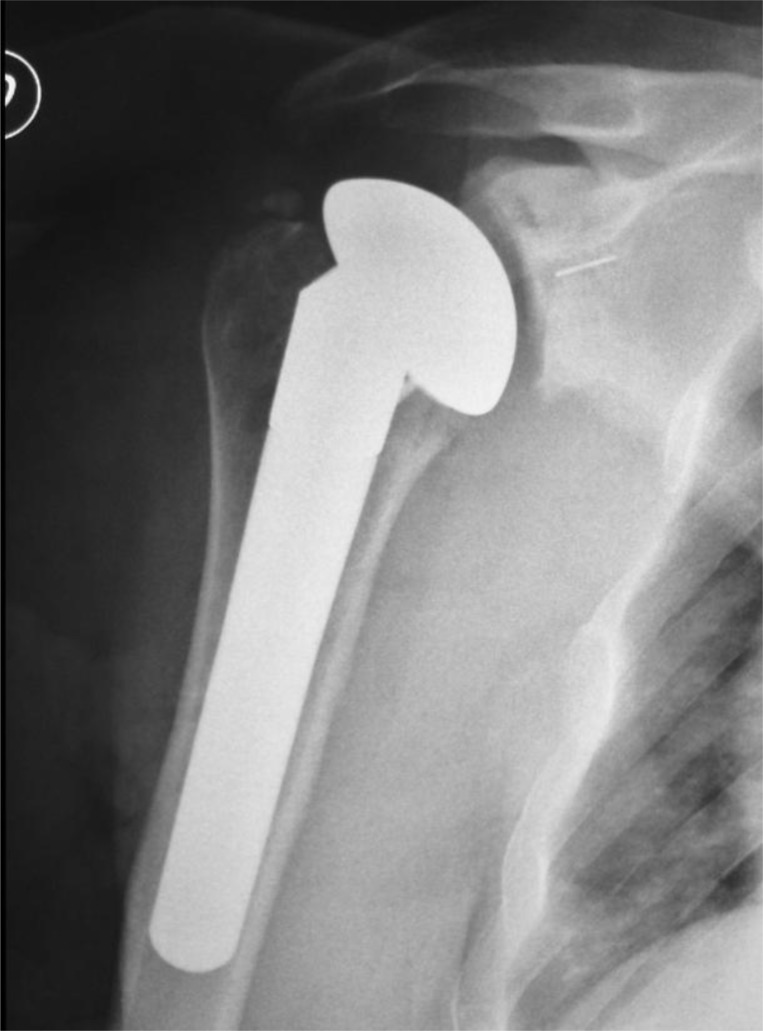

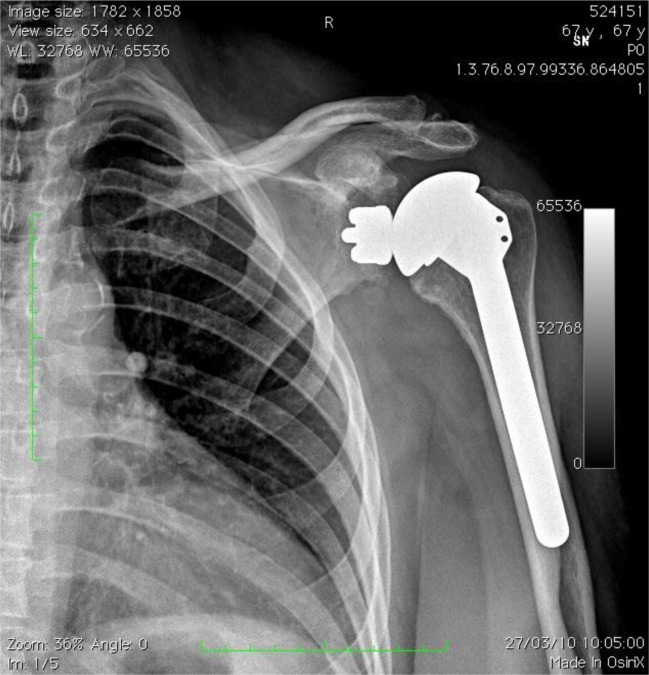

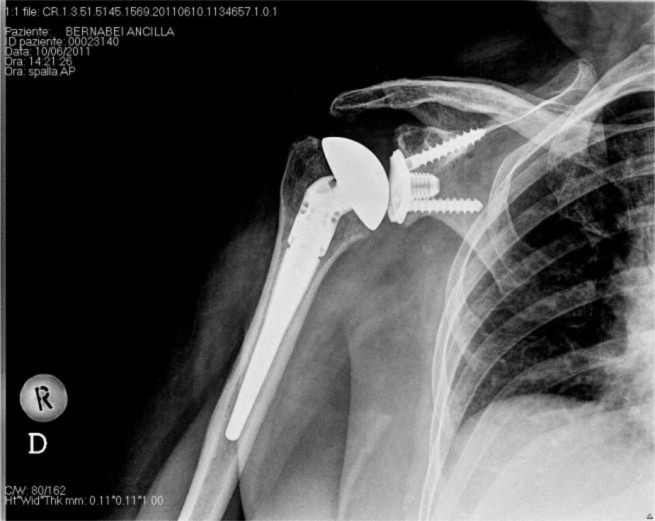

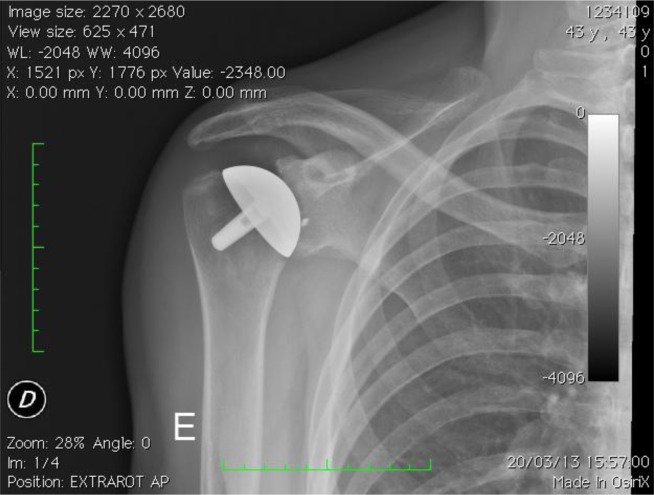

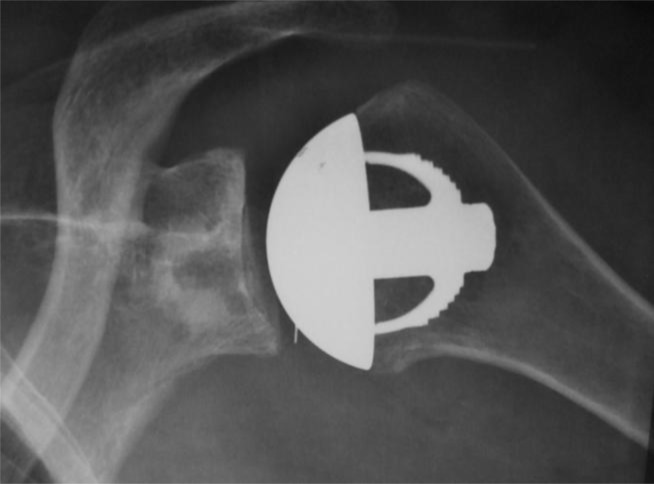
A–F: Postoperative X-ray: cemented stemmed humeral prostheses (A), TSA with uncemented humeral component and cemented all-polyethylene glenoid component (B), TSA with TMT glenoid component (C), TSA with metal-backed glenoid component (D), humeral resurfacing (E), uncemented stemless shoulder prostheses with cemented polyethylene glenoid component (F).

Standard radiographs are performed to evaluate the appropriate prostheses position and stability. Postoperative X-ray of the shoulder prostheses models described in this article are represented in the Figures 25 A–F .

## 4. DISCUSSION

Literature evidence showed that anatomical shoulder arthroplasty provides good results in terms of pain relief and recovery of shoulder function [ 16 , 17 ] with emphasized better clincal outcomes of total arthroplasty than humeral replacement [ 18 – 20 ] . Although hemi shoulder arthroplasty (HAS) is advantageous in selected cases of osteonecrosis and eccentric osteoarthritis [ 19 ] , it represent a challenging option in severe shoulder osteoarthritis for the risk of glenoid erosion [ 21 ] . On the other hand, the weak point in TSA is the loosening of the glenoid component [ 22 – 24 ] , while humeral loosening remain very uncommon [ 14 , 15 ] . Cemented polyethylene glenoid failure gives an account of the unsatisfactory results after TSA [ 23 ] and the modes of failure includes: *1) failure of the component itself* (distortion of the prosthetic surface, fractures or delamination of the component), *2) failure of the component seating* (inadequate preparation of the bone surface, prostheses not fully seated on the prepared bone, loss of cement interposed between the body of the component and the glenoid bone surface, fractures or bony deficience, resorption of bone surface), *3) failure of inizial component fixation* (suboptimal cement technique, fixation in bone of limited quantity and poor quality), *4) failure of bone* (progression of radiolucen lines, immunological response to polyethylene, osteolysis), *5) prosthetic loading* (conforming joint surfaces, rim loading, weight-bearing shoulder prosthesis, glenoid component version, glenohumeral instability, rotator cuff insufficiency).

In order to the glenoid reaming and fixation technique we can explain some considerations: i) adequate seating and stability of the glenoid prosthesis may be in relation to the bone surface changes induced by reaming [ 24 ] ; furthermore glenoid could be not seated due to incomplete removing of the glenoid ostephytes. Cementation can be performed either manually or with a syringe; on this regard, micro-CT scans demonstrated that a syringe achieved circumferential fixation of 100% of pegs compared with only 53% of those fixed with finger pressure [ 24 ] . These findings prompted us to adopt syringe pressurization for glenoid implantation at our institution. Glenoid component fixation may be affected by glenoid mineralization patterns that have been shown to be heterogeneous, with a linear relatonship between bone mineral density and strength distribution. The most common patterns of mineralizations found were typically bicentric, with the highest values detected in squares 4 and 6 of anterior and posterior glenoid [ 25 ] . For these reasons we suggest to perform an accurate preoperative CT analysis to measure bone loss and version and consider bone graft for osseointegration in case with a severe glenoid erosion.

Partially cemented glenoid prostheses with flanged central peg have been advocated due to the potential capacity to favor osseointegration. During this surgical procedure the central peg remain uncemented and the flanges are completely embedded into bleeded cancellous bone (“morselized bone graft”) [ 26 ] .

Although recent studies [ 26 , 27 ] and our CT findings (Merolla G unublished data) showed a good bone mantle around the central uncemented peg, the follow-up is too short to assert the complete bone osseointegration.

Surgical procedure for metal-back glenoid requires a central press-fit into place and fixation with 2 screws that represented a rigid system with polyethylene liner in surface. A flat metal back flash with the glenoid ensure prostheses stability but is at risk for bone resorption around the metallic baseplates and screws [ 28 ] ; furthermore polyethylene wear can induce metal-on-metal contact with associated synovitis

Boileau P et al [ 28 ] in a prospective, double-blind randomized study showed that the survival rate of cementless, metal-backed glenoid components is inferior to cemented all-polyethylene components and the incidence of radiolucency at the glenoid-cement interface with all-polyethylene components was high. Taunton et al [ 29 ] reported a 5 years survival estimate free of revision or radiographic failure of 79.9% and a 10 years survival estimate of 51.9 % with a flat metal-backed bone ingrowth glenoid component. Biomechanical laboratory studies have described high stresses within the polyethylene of metal-backed glenoid components with the implication that these components will have inferior wear properties [ 30 , 31 ] . These biomechanical findings, combined with clinical data [ 29 ] , indicate that the increased stresses due to metal backing increases the polyethylene wear rate and leads to clinical failure in some shoulders. Conversely, Castagna et al [ 32 ] reported good mid-term outcomes using a dual radius metal-backed glenoid, suggesting that the design and the shape of the metal back could affect the results. These authors emphasize the effects of highly stiff and thick metal-backing to give rigidity to the implant with reduced stresses in the polyethylene component and the underlying bone, but at the same time, they highlighted that thicker metal-backing result in higher metal-bone and polyethylene metal interface stresses which may lead to an interface disruption with separation of the component from bone or separation of polyethylene from the metal-backing. As alternative to the stemmed implants, the metallic humeral resurfacng or total shoulder resurfacing with polyethylene glenoid component have become popular, offering benefits for the surgeon and the patients. In fact, retaining the humeral head is easier to maintain the correct version, offset and neck inclination [ 33 , 34 ] , although the glenoid could be difficult to expose and replace because the humeral head is not resecated [ 35 ] . Long term results reported patient satisfaction was 95%, and the survivorship of the humeral prostheses was 96% [ 36 ] . We can consider humeral resurfacing as a viable option in young active patients less than fifty-five years of age, expecting favourable results for pain relief and restore of desired function [ 37 ] . As for stemmed prostheses, glenoid erosion remain the main factor affecting humeral head replacement (38) and recent research findings reported unsatisfactory outcomes using meniscus allograft for glenoid arthroplasty (38).

In order to reduce the risk of glenoid erosion, Merolla et al (38) supported two speculative hypotheses. First, the size should be reduced, favouring small prosthesis covering about the 80% of the head surface and having a head height not exceeding 1.5 mm; second, in those cases with preoperative glenoid arthritis could be reasonable to place the prosthese more valgus to limit the concentric loading of the head prostheses on the glenoid surface which helps to increase the risk of central glenoid erosion. An additional option to conventional arthroplasty is represented by stemless prostheses, that allow to gain an anatomic reconstruction of the proximal humerus, through an automatic centering in the metaphyseal, both in the normal bone structure and in case of poor quality or soft bone structure [ 39 ] . However, when we choose this kind of prostheses, the humeral head cutting must be as accurate as possible to obtain a flat and stable bone surface for a suffcient osseointegration of the implant. The use of short stem humeral component may represent a good future perspective, but clinical and radiographic findings are not yet available for any speculatyve hypothesis.

## Figures and Tables

**Fig. 7:**
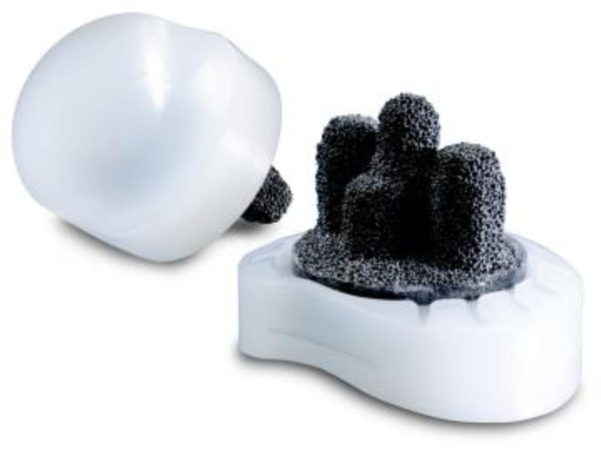
TMT glenoid component (Zimmer, Warsaw, IN - USA).

**Fig. 16:**
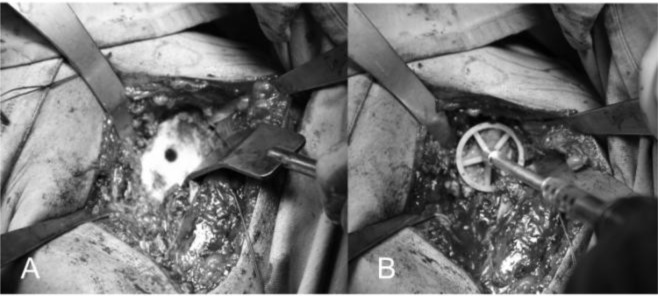
A–B: First central hole (A) and reaming of the glenoid surface (B).
